# Identifying work related injuries: comparison of methods for interrogating text fields

**DOI:** 10.1186/1472-6947-10-19

**Published:** 2010-04-07

**Authors:** Kirsten McKenzie, Margaret A Campbell, Deborah A Scott, Tim R Discoll, James E Harrison, Roderick J McClure

**Affiliations:** 1 National Centre for Health Information Research and Training, Queensland University of Technology, Victoria Park Road, Kelvin Grove, Queensland, 4059, Australia; 2 Queensland Injury Surveillance Unit (QISU), Mater Hospital, Stanley Street, Brisbane, Queensland, 4101, Australia; 3 School of Public Health, University of Sydney, Fisher Road, Camperdown, New South Wales, 2050, Australia; 4 Research Centre for Injury Studies, Flinders University, Laffer Drive, Bedford Park, South Australia, 5042, Australia; 5 Monash University Accident Research Centre, Monash University Clayton Campus, Melbourne, Victoria, 3800, Australia

## Abstract

**Background:**

Work-related injuries in Australia are estimated to cost around $57.5 billion annually, however there are currently insufficient surveillance data available to support an evidence-based public health response. Emergency departments (ED) in Australia are a potential source of information on work-related injuries though most ED's do not have an 'Activity Code' to identify work-related cases with information about the presenting problem recorded in a short free text field. This study compared methods for interrogating text fields for identifying work-related injuries presenting at emergency departments to inform approaches to surveillance of work-related injury.

**Methods:**

Three approaches were used to interrogate an injury description text field to classify cases as work-related: keyword search, index search, and content analytic text mining. Sensitivity and specificity were examined by comparing cases flagged by each approach to cases coded with an Activity code during triage. Methods to improve the sensitivity and/or specificity of each approach were explored by adjusting the classification techniques within each broad approach.

**Results:**

The basic keyword search detected 58% of cases (Specificity 0.99), an index search detected 62% of cases (Specificity 0.87), and the content analytic text mining (using adjusted probabilities) approach detected 77% of cases (Specificity 0.95).

**Conclusions:**

The findings of this study provide strong support for continued development of text searching methods to obtain information from routine emergency department data, to improve the capacity for comprehensive injury surveillance.

## Background

Work-related injuries in Australia are estimated to cost around $57.5 billion annually [1], however there are currently insufficient surveillance data available to support an evidence-based public health response [2]. Work-related injury surveillance data are obtained from various disparate data sources, with each source capturing different samples, populations, injury types, severity levels and injury causation elements. The main sources in Australia include: mortality data, hospital morbidity data, special purpose injury-specific data collections and workers compensation claim data.

Mortality data provide a national standardized source of injury information, with additional detail available from the National Coroners Information System, however only a small proportion of work-related injuries have fatal outcomes. Hospital morbidity data provides a national standardized source of injury information, with 'Activity at the time of injury' (referred to as 'Activity code' for remainder of paper) coded routinely and including a category 'while working for income. This data source also includes an item flagging the cases funded by workers' compensation insurance [3]. However, the quality of documentation of these items in the medical records is not validated, the quality of coding of activity is variable, and hospitalization data only captures the more severe injury cases. Special purpose injury-specific data collections include those focusing on the injury event details, and those recording details of injury, clinical intervention and injury outcome. Injury surveillance emergency department-based data collections focusing on injury event information are only available in Queensland and Victoria for a sample of hospitals, and rely on ascertainment of cases and completion of injury surveillance forms by triage nurses, with work-related injuries flagged in an Activity code. Clinical registries of severe injury cases (i.e. trauma registries), with complete ascertainment of all severe injuries presenting to hospitals within specified populations are focused on trauma system performance and mostly record little information regarding injury causation. Some registries include an Activity code which assists in the ascertainment of the proportion of work-related injuries in the registry samples. Workers' compensation claim data contains details of injured persons who have had a workers' compensation claim accepted. This excludes those individuals who are injured but who do not make a claim, who were unsuccessful in their claim, or who weren't eligible for workers' compensation (such as self employed, work experience etc). Workers compensation databases in Australia have been found to underestimate the number of work-related cases by up to 65% [2,4-6].

In addition to these usual sources of data for injury surveillance, routine emergency department information provides an opportunity to increase the comprehensiveness of the injury surveillance systems. The information, however, lies in the free text fields completed by the triage nurse when recording the patients presenting complaint. In Australia, this free-text emergency department (ED) data has been collected in an electronic format over several years in many hospitals. ED data collections have been subject to review and some improvements have been made over the last few years at a national level to enhance the amount of available detail about ED presentations, with the development of a national minimum dataset prescribing standardized data collection protocols for ED data for tertiary and large hospitals from 2003/04 [7].

However, this source currently has very limited value for injury surveillance due to the lack of data items on diagnosis and reason for attendance, including external cause.

ED presentation data from injury surveillance collections in Queensland and Victoria have been assessed previously as a source of surveillance information on work-related injuries, and injury description text was reported to be potentially useful [8]. The extent to which text fields can be used for this purpose has not been explored.

Despite the acknowledgement that injury narrative data may contain valuable information, they remain underutilized due to difficulties in analyzing and interpreting free text data sources [9]. A systematic review of the literature on the use of narrative text for injury surveillance found that a variety of methods have been used to extract and translate text data into a useable form for injury surveillance, though few studies described methods in detail, and less than half of the studies described quality assurance methods to validate the findings (such as reference to sensitivity, specificity and positive predictive value estimates) [10]. The three main approaches which have been used for interrogating text data can be summarised as being: Manual review and coding, text search methods, and automated or semi-automated methods employing statistical and/or lexical software tools using Bayesian clustering principles.

Previous studies using text interrogation methods to examine work-related injuries include some in which each of these approaches has been used; ie manual coding and review [11-18], keyword searches [19-21], a combination of manual coding and keyword search algorithms [22-26], structured abstraction tools and manual coding [27-30], or more complex statistical Bayesian clustering approaches [31]. The focus of these papers has been either to enable the identification of cases where coded fields were insufficient or unavailable for case identification, and/or to identify the circumstances of injury events where coded fields were insufficient or unavailable for detailed circumstance information.

No workplace injury study to date has compared the results obtained from each of the different text search methods. If text searching is to be used to increase the injury surveillance information by enabling use of routine emergency department datasets, then it is essential to quantify the performance of the different methods. The purpose of this study was to compare methods for interrogating text fields for identifying potential work-related injuries presenting at EDs in Queensland to inform future surveillance of work-related injury using narrative text. The specific aims were to:

1. Describe and compare different text interrogation methods for identifying potential work-related injury cases.

2. Examine sensitivity, specificity, and positive predictive value of each text interrogation method against the coded injury surveillance Activity code meaning injured while 'Working for an income'.

## Methods

### Data source

Queensland Injury Surveillance Unit (QISU) data from 2002-2007 was utilized for this study. This dataset contains injury surveillance information collected from persons presenting with an injury or, in the case of children, from the accompanying adult, for a sample of 14 ED's in Queensland. The QISU data are obtained from hospital emergency departments, which chiefly treat patients in the acute phase of injury, soon after occurrence. As this study utilised secondary de-identified data, the study was considered exempt from ethics review by the university human research ethics committee.

QISU is designed to capture injury surveillance data from the National Data Set for injury surveillance (NDS-IS) [32] to inform injury prevention policy development and relevant health promotion initiatives and for advocacy to support legislative and policy change. The NDS-IS is compatible with the International Statistical Classification of Diseases and Related Health Problems (ICD) which is used internationally for morbidity and mortality coding, using the same basic categories but providing some extensions of categories in place and activity to provide greater specificity for injury surveillance. QISU data is collected by the triage nurse in the ED during the triage process. When a patient presents for treatment of an injury the triage nurse ticks a box that says 'Injury yes or no'. If she ticks yes she is prompted to complete an injury surveillance screen. The demographics and the presenting problem are auto-populated from the admission screen (EDIS system) and the nurse then completes the injury information. This information includes the cause of injury, nature of injury, where the injury occurred, what the activity of the injured person was at the time, the object or substance involved in the injury and the role of human intent. QISU data contains both coded information and free text fields that have the potential to provide useful information regarding whether the injury was work-related and about the nature of the work and injury event. The Activity code is assigned to indicate what the person was doing at the time of the injury event. This variable has a range of codes, though for the purposes of this study the Activity codes were summarized as either 'Working for an Income' (referred to throughout this paper as 'Work Activity code') or 'Other Activity code' (combining all other codes besides the 'Working for an Income' code). The Injury Description text field is a short free text field (maximum 255 characters) to capture the details regarding the circumstances surrounding the injury event.

### Text interrogation methods

Three approaches were used to interrogate the Injury Description text field for this study: a basic keyword search, a detailed index search, and a content analytic text mining approach. As this study aimed to identify 'potential' work-related cases from information provided in the text field, the presence of target words/phrases/concepts was considered sufficient for flagging a case as being 'potentially' work-related. The presence of a negation phrase, such as 'not working' or 'not work-related', was not used to rule cases out of consideration because such terms can be used when describing work-related cases (e.g. 'injured hand on saw that was not working properly'; 'hurt back at work yesterday and did not work today', etc). Hence, including restrictions in the first round of case selection may exclude potential cases of interest. This study aimed to maintain a balance between specificity and sensitivity to ensure criteria for inclusion was not too strict to miss a lot of cases (i.e. sensitivity), but not too broad to include a lot of cases which were not likely to be work-related cases (i.e. specificity). Thus, the approach to case selection described in this paper may be tightened using a stricter case selection criterion if the objectives of the study require greater specificity.

#### 1. Keyword search

The first approach to interrogating the text data was to conduct a simple keyword search using the word 'work' and key phrases to identify cases describing the activity of working for an income at the time of the injury. Cases were flagged as being potentially work-related if they contained the word 'work' (which included variations such as 'work-related', 'worker' etc) and these cases were used in the sensitivity/specificity analysis.

#### 2. Index search

The second approach to interrogating the text data was to conduct a keyword search using a detailed index developed from a manual review of sampled cases. A random sample of 1000 cases coded with a 'Work Activity code' but which did not contain the word 'work' were extracted for manual review to examine the words other than 'work' that were used to describe work-related activities. The sample size of 1000 cases was chosen to provide sufficient numbers to gather a broad range of terms for the creation of an 'index' whilst still remaining manageable to review manually. A qualified Health Information Manager (HIM) read each of the text descriptions and, using an Excel spreadsheet, manually extracted the keywords used within the text to describe the injury event under the following headings: nature of injury, body site, activity task, precipitating mechanism, contributory factor, injury event/exposure, object involved, physical force involved, substance involved, human agency involved, organism involved, work location, occupation, industry, nature of work, and safety/preventative devices used.

The frequency of keywords from the activity, object, location, occupation and safety/preventive devices were explored as these elements provided the most work-specific terms out of all elements that were extracted. There was considerable overlap in a number of terms, for example terms such as 'weld' could be present as an activity ('welding'), or an object or occupation ('welder'). Terms from all lists were combined and sorted and the smallest word-stems which could be used in an index search algorithm identified. Terms recorded less than five times were excluded (to ensure infrequently used terms were not included in the index making the index too large to be unwieldy for general use) and a final index list of 50 terms was created for use in an index search algorithm which was applied to the full dataset. Cases which contained any of these keywords were flagged for review in the specificity/sensitivity analysis phase. These terms were then combined with cases flagged in the keyword search to identify whether the sensitivity of detection of cases could be improved by using either the word 'work' or any of the index terms to identify 'Work Activity' cases.

#### 3. Content analytic text mining approaches

The third approach used content analytic text mining software (Leximancer [33,34]) to categorise cases to the two Activity groups based on the terms recorded in text. Leximancer software examines the clustering of terms, based on the frequency and co-occurrence of text within sentences and, using terms identified in the text string, identifies the broad concepts captured in each text string. Leximancer software identifies the high frequency/highly co-occurring terms and concepts which appear with each of the actual coded Activity groups, and then assigns a probability of group membership to each case based on the mixture of terms and concepts identified from the text string.

As Leximancer has not been widely used in this domain before, the process used in this study is described in detail below:

a) Preprocessing - A comma separated file with File ID, binary Activity code (0 = Other Activity code, 1 = Work Activity code) and Injury Description was imported into Leximancer. The settings that were applied in the pre-processing phase were as follows:

• The whole Injury Description text segment was examined in one block per case to identify the broad concepts described for each case and there was no requirement for the presence of grammatical sentences (i.e. prose test threshold set to 0)

• Grammatical variants of a word were represented by a single term (e.g. work, works, working were represented by the one term 'work')

• The default 'stop-list' (the list of frequently occurring words, such as 'and', 'the' and 'but' which hold little or no semantic information and are excluded from frequency/co-occurrence statistics) was modified to retain for analysis words that might refer to body parts (e.g. back, face, feet), mechanisms of injury (e.g. cut) or injurious objects (e.g. saw).

b) Automatic Concept Identification (all default settings used) - Automatic concept identification was selected to enable the software to identify concepts from the text and all concepts were retained for the exploratory analysis to enable the software to automatically assign terms to concepts.

c) Thesaurus Learning - The following settings were applied in this phase:

• 'Concept generality' was set to 10 to ensure extracted concepts were not too broad

• 'Learn from Tags' was selected so that the software searched for concepts associated with each of the two coded Activity groups (or 'Tags') independently

• 'Number of concepts to discover' was turned off to allow the software to identify the number of relevant concepts (default setting)

• The themed discovery option of 'Concepts in each' was requested to identify concepts which discriminated the two coded Activity groups (i.e. concepts that were more strongly associated with one of the Activity groups and not the other, to enable identification of the words and concepts associated with the 'Work Activity code' compared to the 'Other Activity code'.

d) Locate Concepts - All concepts identified by the software were selected to be included on the Leximancer concept map (default setting). The two 'Activity' groups generated by the software were included on the concept map. These software generated 'Activity' groups are referred to as 'SPV Tags' in the software, or 'Supervised Tags', as they are classified after learning from the common terms and concepts identified from each coded Activity group (more detail provided in approach number 3 below). These will be referred to as the 'Work SPV Tag' and the 'Other Activity SPV Tag' in this paper. All cases assigned to the 'Work SPV Tag' were flagged and merged with the full SPSS dataset for calculation of sensitivity and specificity.

Two techniques were used to examine the sensitivity and specificity of the content analytic text mining generated 'SPV Tags'. The first technique simply accepted all cases assigned the 'Work SPV Tag' as being 'Work Activity' cases for calculation of sensitivity and specificity. However, as several concepts can be assigned to each text description, a case may be assigned both SPV Tags (i.e. both 'Work SPV Tag' and 'Other Activity SPV Tag') with different probabilities of group membership depending on the concepts identified. For example, a text description stating 'Went home from work after getting piece of metal in eye; eye red and inflamed' would have the concepts 'home' 'work' 'metal' and 'eye', and hence may be assigned a high probability of belonging to the 'Working for an income' group based on the concepts 'work', 'metal', and 'eye' (all common words associated with work-related injuries) and a small probability of belonging to the 'Other Activity' group based on the concept 'home'.

To improve the specificity of case detection, a second technique to examining the sensitivity and specificity of the 'SPV Tags' generated by content analytic text mining made use of the probabilities of group membership to assign cases to Activity groups:

1. Cases with a greater than 0 probability of belonging to the 'Work SPV Tag' and a 0 probability of belonging to the 'Other Activity SPV Tag' were classified as 'Work Activity'.

2. Cases with a greater than 0 probability of belonging to the 'Work SPV Tag' and a greater than 0 probability of belonging to the 'Other Activity SPV Tag', where the probability of belonging to the 'Work SPV Tag' was higher than or equal to the probability of belonging to the 'Other Activity SPV Tag' were classified as 'Probable Work Activity'.

3. Cases with a greater than 0 probability of belonging to the 'Work SPV Tag' and a greater than 0 probability of belonging to the 'Other Activity SPV Tag', where the probability belonging to the 'Work SPV Tag' was less than the probability of belonging to the 'Other Activity SPV Tag' were classified as 'Probable Other Activity'.

4. Cases with a greater than 0 probability of belonging to the 'Other Activity SPV Tag' and a 0 probability of belonging to the 'Work SPV Tag' were classified as 'Other Activity'.

Cases were then reviewed in terms of the proportion of cases coded with a 'Work Activity code' and 'Other Activity code' in each probability assigned Activity group, to identify potentials for modifying the probability cut-offs to strengthen the specificity of case detection.

### Sensitivity/specificity analysis

Sensitivity and specificity were calculated for all text interrogation approaches which involved examining cases flagged as doing a 'Work Activity'/'Other Activity' using each of the techniques within the three broad approaches and comparing these cases to cases coded with a 'Work Activity code'/'Other activity code' using the Activity coded data. The Activity codes were used as the standard against which alternative approaches were measured for the calculation of sensitivity, specificity, and positive predictive value (PPV).

## Results

Overall, of the 208,291 cases recorded in the QISU dataset for 2002-2007, there were 21,419 cases (10.3%) coded as 'Working for an income' using the Activity code and 23,991 cases (11.5%) with an unspecified activity (10 cases were missing an activity code and these cases were excluded from further analysis). Table 1 summarises the results of the text based search techniques (keyword and index) and Table 2 summarises the results of the content analytic text mining techniques.

**Table 1:** Activity codes by text search methods

	Work Activity code		Other Activity code		
Term Search Methods	n	%	n	%	Total
Keyword search					
'Work' in text string	12,457	58.16	1,916	1.03	14,373
'Work' not in text string	8,962	41.84	184,946	98.97	193,908

Index term search					
Work index term in string	13,252	61.87	23,434	12.54	36,686
Index term not in string	8,167	38.13	163,428	87.46	171,595

Keyword OR index term search					
Index or keyword in string	17,004	79.4	24,416	13.1	41,420
No index or keyword in string	4,415	20.6	162,446	86.9	166,861

Total	21,419	100	186,862	100	208,281

**Table 2:** Activity codes by content analytic text mining approaches

	Work Activity code		Other Activity code		
Content Analytic Text Mining Methods	n	%	n	%	Total
Binary classification					
'Work SPV Tag'	17,299	80.8	13,894	7.4	31,193
Not 'Work SPV Tag'	4,120	19.2	172,968	92.6	177,088

Adjusted probability classification					
Classified as 'Work Activity'	16,424	76.7	8,699	4.7	25,123
Classified as 'Other Activity'	4,995	23.3	178,163	95.3	183,158

Total	21,419	100	186,862	100	208,281

### Keyword search

Using a basic keyword search for 'work', 14,373 potential 'Work Activity' cases were identified, with 12,457 of these true cases and 1,916 false positives (See Table 1). The sensitivity was 0.58 and the specificity was 0.99.

### Index Search

There were 8,962 cases where there was a 'Work Activity' code but the text did not mention the word 'work'. A random sample of 1000 of these cases was manually reviewed as described in the methodology and an index list created from relevant terms. Of the sample of 1000 cases coded with a 'Work Activity' code, 522 cases had one or more of the 50 index terms present in the injury description. These index terms were then used in an index search on the full QISU dataset.

Using the index search (without including the word 'work' in the search algorithm, 36,686 potential 'Work Activity' cases were identified, with 13,252 of these true cases and 23,434 false positives. The sensitivity was 0.62 and the specificity was 0.87. The most specific terms (i.e. where more than 80% of cases were coded with a 'Work Activity code') were job site (189 cases out of 197 with this term present were coded with a 'Work Activity code'), factory (144/152), labour (48/54), forklift (148/172), mine (1567/1842), construction (246/303), and client (133/165).

When combining the cases flagged as having an index term present or cases flagged with having the word 'work' present, there was an improvement in the sensitivity. Using the index search or the keyword search, 41,420 potential 'Work Activity' cases were identified, with 17,004 of these true cases and 24,416 false positives.

### Content Analytic Text Mining Approaches

Using the content analytic text mining approach with the binary classification (i.e. belonging to the 'Work SPV Tag' or the 'Other Activity SPV Tag'), 31,194 potential 'Work Activity' cases were identified, with 17,299 of these true cases and 13,894 false positives. The sensitivity was 0.81 and the specificity was 0.93.

To improve the specificity of case detection (but potentially decrease the sensitivity), we used the probabilities of group membership to classify cases as into 'Work Activity', 'Probable Work Activity', 'Probable Other Activity' and 'Other Activity'. Cases classified using this approach were then examined in terms of the proportion with a 'Work Activity code' and an 'Other Activity code'.

Examining the proportion of each group with a 'Work Activity code' and an 'Other Activity code', it was found that a large proportion of cases classified as 'Work Activity' or 'Probable Work Activity' (85%) were coded with a 'Work Activity code'. However, 5609 of the 17271 cases (32%) assigned as 'Probable Other Activity' were coded with a 'Work Activity code'. Using this strict probability cutoff reduced the sensitivity of case detection of Work Activity cases (including 'Work Activity' and 'Probable Work Activity') to 0.55. As such, further analysis of the distribution of probabilities by Activity codes for the 'Probable Other Activity' group were examined to identify whether a less strict inclusion cut-off could be used to increase the sensitivity of case detection.

For the 'Probable Other Activity' group, the average and standard deviation of probability differences (i.e. the probability of belonging to the 'Work SPV Tag' minus the probability of belonging to the 'Other Activity SPV Tag') was examined for each Activity code. There was a significant difference between groups with an average probability difference of -1.47 (SD .92) for cases with an 'Other Activity code', and an average probability difference -0.83 (SD .63) for cases with a 'Work Activity code' (T(1,17269) = -47.23, p < 0.01).

Thus, to improve the sensitivity of detection of cases, the criteria for assigning cases to 'Probable Work Activity' and 'Probable Other Activity' were modified as follows. All cases coded as 'Work Activity', 'Probable Work Activity' and 'Other Activity' were classified using the same classification approach described in the method section. However, cases originally coded as 'Probable Other Activity' using the classification approach described in the method section were recoded as 'Probable Work Activity' if the probability difference was less than 1.47. The value of 1.47 was chosen as the cut-off point as it is the average probability difference for cases with a 'Work Activity code' plus one standard deviation.

Examining the proportion of each group with a 'Work Activity code' and an 'Other Activity code', it was found that cases coded with a 'Work Activity code' in the 'Probable Other Activity' group decreased substantially. However, there was a corresponding increase in the number of cases coded as 'Probable Work Activity' where an 'Other Activity code' was assigned.

Using the content analytic text mining approach with adjusted probability classification, 25,123 potential 'Work Activity' cases were classified, with 16,424 of these true cases and 8,699 false positives (See Table 2). The sensitivity was 0.77 and the specificity was 0.95.

### Comparison of text interrogation methods

**Figure 1:**
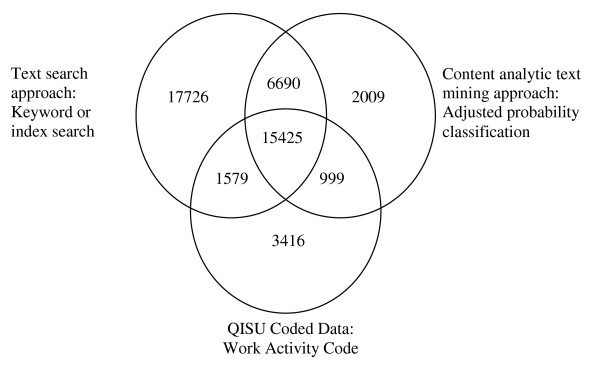
**Identification of cases of work-related injuries using 'Work Activity' code, a text search approach and a content analytic text mining approach**.

A summary of the findings from each approach is shown in Table 3 including sensitivity, specificity, numbers of true cases and false positives and PPV) and a comparison of the cases identified using the text search approach to the final content analytic approach and the 'Work Activity code' are shown in a Venn diagram in Figure 1. While the basic keyword search produced the poorest sensitivity of all approaches, the PPV for this approach surpassed all other approaches with only 1916 false positives identified, compared to 12,457 true cases. In comparison, the content analytic approach with adjusted probability, had a higher sensitivity at 0.77 and had the second highest PPV (at 0.65), though this represented 8,699 false positives and 16,424 true cases.

**Table 3:** Summary of case identification results using each method

Approach	Number of true cases identified	Number of false positives	Sensitivity	Specificity	PPV
Text Search Approaches					
Basic keyword search	12,457	1,916	0.58	0.99	0.87
Index search	13,252	23,434	0.62	0.87	0.36
Keyword OR index	17,004	24,416	0.79	0.87	0.41
Content Analytic Approaches					
Binary classification	17,299	13,894	0.81	0.93	0.55
Adjusted probability classification	16,424	8,699	0.77	0.95	0.65

## Discussion

This study aimed to compare different approaches for interrogating text fields for identifying work-related injuries presenting at EDs in Queensland to inform future surveillance of work-related injury using narrative text. Three approaches were compared: a basic keyword search, an index search using common work terms identified by a manual review, and a more complex content analytic text mining approach. Each approach was examined and refined using various techniques, and the final sample of cases identified using each approach were compared in terms of sensitivity, specificity, and positive predictive value of each approach.

Any system used to identify cases needs to have good sensitivity (to identify true cases) and good specificity (to avoid including non-cases). However, there is a trade-off between the two, with one likely to decrease as the other increases [10,25]. For case identification, it is usually more important to have a sensitive system. This allows nearly all cases to be identified, but at the cost of including some non-cases. However, non-cases then need to be identified and excluded through a check of the initial identified 'cases'. In contrast, a specific system would mean few non-cases would be initially included but those cases that are missed would probably not be identifiable through other means. However, in exchange for high specificity, sensitivity may be compromised. Therefore, sensitivity calculations were provided as an estimate of the likely under-enumeration of cases using each search strategy, to enable studies focused on making incidence-type assessments to make adjustments for the extent of likely underestimation. Specificity calculations were provided as an estimate of the likely inclusion of false cases, to enable studies focused on describing the epidemiology of specific types of injuries for specific groups to be aware of the extent of manual review and exclusion which may be required. Positive predictive values of each of the approaches were also provided as the likely number of false positives is an important consideration given the small proportion of work-related injuries within the substantial number of non-work-related injuries that are likely in emergency department presentation data.

A simple keyword search on work had the highest specificity 99% of all search methods, and it was simple to replicate using any software which allows text search and flagging. If seeking to identify a small sample of true cases for further audits/follow-up/linkage studies, the use of a simple keyword search may be appropriate given the high specificity of this approach. However, the sensitivity of the keyword search was relatively low, detecting only 58% of cases coded with a 'Work Activity code'. Hence, if seeking to enumerate incidence figures, the basic keyword approach would seriously underestimate cases.

Using an index search to extend the basic keyword search improved the sensitivity to 0.79, but reduced the specificity to 0.87, identifying a large number of false positives. Therefore, considerable review of identified cases would be required if undertaking this approach to remove false positives from identified cases. Furthermore, the development of the index through the manual review and identification of key terms was considerably resource intensive.

The content analytic text mining approach using adjusted probabilities provided a balance of sensitivity and specificity. The.approach showed improvement in terms of sensitivity compared to the basic key word search, detecting 77% of cases coded with a 'Work Activity code' compared to 58%. Specificity of the content analytic text mining approach was somewhat lower than for the basic keyword method (0.95 vs 0.99), and this is reflected in the lower PPV. Sensitivity was similar to that obtained by the combined keyword and index search, though the specificity was improved (0.95 for content analytic text mining and 0.87 for keyword/index search). This improved specificity derived from the software's ability to discriminate the high frequency terms associated with work-related cases compared to non-work-related cases. In addition to identifying and extracting relevant terms the software also provided a conceptual map of the circumstances and types of injury events associated with work-related compared to non-work related cases as well as a detailed list of common terms and concepts associated with 'Work Activity' cases. This additional information provides an enhanced insight into the injury events beyond case identification purposes. However, this process does require the use and understanding of specialist content analytic software.

All of these approaches rely on the quality of the information recorded in the text field. Where there was no information or limited information recorded, none of the text interrogation approaches were able to identify cases effectively. While well structured and detailed injury descriptions recorded in the text field at triage would provide ideal data for use in content analytic text mining approaches, often such structure and detail is not possible in a busy emergency department environment. However, the inclusion of key terms and short phrases in the text description to describe the injury circumstances (instead of no information or limited information), would still enable better end-use of these data. None of these approaches rely on grammatical prose to enable case detection, and misspellings and abbreviations are also able to be accommodated by each approach provided some information is recorded in the text description.

This study used the Activity code assigned during the triage process as the standard against which alternative methods were measured for identification of 'Work Activity' cases. However, there has been limited systematic validation of the accuracy of these coded Activity data, and hence some of the 'false positives' detected may in fact be true cases previously undetected/not coded at triage. To avoid the subjective biases inherent when reviewing secondary text data to assign a case as work-related or not, the work activity code was used as the standard for measurement because a) those assigning this code had source documentation and the patient present when they assigned the code; b) the text field is routinely reviewed by QISU coders to validate the accuracy of coded data; and c) this code is relied on by other users of the data as the indicator of an injury being work-related. Further research could be conducted on the cases identified via each of these text interrogation methods to audit the 'false positives' via more comprehensive medical record review to validate the quality of the coded Activity data (and provide further evidence to strengthen the text interrogation approaches).

This study relied on data collected as part of an injury surveillance system operating in a sample of emergency departments in Queensland Australia (QISU), and it is important to consider the generalisability of these findings to other systems. QISU follows the guidelines recommended by the World Health Organisation for injury surveillance. The method for collection (i.e. in emergency departments by triage nurses), injury causation classification (i.e. the NDSIS which is compatible with the ICD), the collection of a short free-text description of the injury event, and the quality assurance approaches (i.e. review of data by QISU coders to validate codes), are all comparable with other major surveillance systems both nationally (such as VISU) and internationally (such as the NEISS in the USA, CHIRPP in Canada, EUIDB in Europe, and the HASS/LASS in the UK). As such, the findings from this study are relevant to other similar surveillance systems in terms of approaches for interrogating text data, with similar issues regarding the use of text data identified by researchers using these other systems [10]. The findings may also be useful for individuals using routinely collected text fields, particularly where there is no or limited coded Activity data (such as in the 'presenting problem' field often collected in emergency department data collections). While it is possible that special injury surveillance collections may be more likely to record more detailed text regarding the injury event, there is limited evidence available to validate this assumption. Furthermore, the approaches for interrogating text data (even scant descriptions) have applicability across systems regardless of the comprehensiveness of the text, though more detailed descriptions would certainly enable more complete ascertainment of cases.

## Conclusions

There has been limited research conducted to evaluate the quality, accuracy and completeness of injury causation information recorded in emergency department text fields, or to develop approaches to the standardization of text entry. As the majority of emergency department data available in Australia does not have an Activity code available to identify potential work-related cases (beyond special injury surveillance sites in Queensland and Victoria), but does have text fields available for presenting problems, evaluating, developing and standardizing these text data would enable better use of these data for injury monitoring purposes.

The findings of this study provide strong support for continued development of text searching methods to obtain information from routine emergency department data, to improve the capacity for comprehensive injury surveillance. The effectiveness of text searching depends both on the technical capabilities of the software and on the extent to which the text has the required information to address the problem under investigation.

## Competing interests

The authors declare that they have no competing interests.

## Authors' contributions

KM contributed to the conceptual design of the manuscript, and was responsible for conducting the literature review, writing the first draft of the manuscript, compiling all authors responses, and preparing the final version of the manuscript. MC assisted with the coding and analysis of the data and reviewed and commented on each draft of the manuscript. DS contributed to the conceptual design of the manuscript, assisted with the literature review and reviewed and commented on each draft of the manuscript. TD provided context to the manuscript in terms of occupational injury research, and reviewed and commented on each draft of the manuscript. JH contributed to the conceptual design of the manuscript, and provided context to the manuscript in terms of injury surveillance implications and reviewed and commented on the final draft of the manuscript. RM contributed to the conceptual design of the manuscript, provided context to the manuscript in terms of injury prevention implications, and reviewed and commented on each draft of the manuscript. All authors read and approved the final manuscript.

## Pre-publication history

The pre-publication history for this paper can be accessed here:

http://www.biomedcentral.com/1472-6947/10/19/prepub

## References

[B1] Australian Safety and Compensation Council The Cost of Work-related Injury and Illness for Australian Employers, Workers and the Community: 2005-06 2009 Canberra: Australian Government http://www.safeworkaustralia.gov.au/swa/AboutUs/Publications/CostofWork-relatedInjuryandIllness.htm

[B2] Driscoll T Mitchell R Mandryk J Healey S Hendrie L Hull B Coverage of work related fatalities in Australia by compensation and occupational health and safety agencies Occup Environ Med 2003 60 195 200 10.1136/oem.60.3.195 12598667 PMC1740488

[B3] Harrison J Flood L Driscoll T Work-related injury hospitalisations Australia: 2002-03 and 2003-04 2007 Canberra: Australian Safety and Compensation Council

[B4] Mitchell R McClure R Driscoll T Refining estimates of hospitalised work-related injury in NSW, 2000-01 to 2004-05 Australian & New Zealand Journal of Occupational Health and Safety 2008 24 33 42

[B5] Muscatello D Mitchell R Identifying work-related injury and disease in routinely collected NSW hospitalisation data NSW Public Health Bulletin 2001 12 195 8 10.1071/NB01066 12105574

[B6] Mitchell R Williamson A Examining the burden of work-related hospitalized injuries: definitional issues Injury Prevention 2008 14 2 101 5 10.1136/ip.2007.017442 18388230

[B7] Australian Institute for Health and Welfare National non-admitted patient emergency department care data collection 2009 http://www.aihw.gov.au/hospitals/napedc_database.cfm [updated 2009; cited 28th May 2009];

[B8] Driscoll T Harrison J Work-related injury emergency department presentations, 2002-03 and 2003-04 2007 Canberra: Australian Safety and Compensation Council

[B9] Smith GS Public health approaches to occupational injury prevention: do they work? Injury Prevention: Journal Of The International Society For Child And Adolescent Injury Prevention 2001 7 Suppl i3 10 11565968 10.1136/ip.7.suppl_1.i3 PMC1765411

[B10] McKenzie K Scott D Campbell M McClure R The Use of Narrative Text for Injury Surveillance Research: A Systematic Review Accident Analysis and Prevention 2010 in press (Accepted 25/09/2009). doi:10.1016/j.aap.2009.09.020. 20159054 10.1016/j.aap.2009.09.020

[B11] Bentley TA Parker RJ Ashby L Moore DJ Tappin DC The role of the New Zealand forest industry injury surveillance system in a strategic ergonomics, safety and health research programme Applied Ergonomics 2002 33 5 395 403 10.1016/S0003-6870(02)00037-6 12236648

[B12] Bentley TA Parker RJ Ashby L Understanding felling safety in the New Zealand forest industry Applied Ergonomics 2005 36 2 165 75 10.1016/j.apergo.2004.10.009 15694070

[B13] Bulzacchelli MT Vernick JS Sorock GS Webster DW Lees PS Circumstances of fatal lockout/tagout-related injuries in manufacturing American Journal Of Industrial Medicine 2008 51 10 728 34 10.1002/ajim.20630 18702095

[B14] Fordyce TA Kelsh M Lu ET Sahl JD Yager JW Thermal burn and electrical injuries among electric utility workers, 1995-2004 Burns 2007 33 2 209 20 10.1016/j.burns.2006.06.017 17116371

[B15] Hendricks KJ Layne LA Adolescent occupational injuries in fast food restaurants: an examination of the problem from a national perspective Journal Of Occupational And Environmental Medicine/American College Of Occupational And Environmental Medicine 1999 41 12 1146 53 10609237 10.1097/00043764-199912000-00021

[B16] Kemmlert K Lundholm L Slips, trips and falls in different work groups--with reference to age and from a preventive perspective Applied Ergonomics 2001 32 2 149 53 10.1016/S0003-6870(00)00051-X 11277507

[B17] Husberg BJ Fosbroke DE Conway GA Mode NA Hospitalized nonfatal injuries in the Alaskan construction industry American Journal Of Industrial Medicine 2005 47 5 428 33 10.1002/ajim.20158 15828070

[B18] Lipscomb HJ Dement JM Behlman R Direct costs and patterns of injuries among residential carpenters, 1995-2000 Journal Of Occupational And Environmental Medicine/American College Of Occupational And Environmental Medicine 2003 45 8 875 80 12915789 10.1097/01.jom.0000083035.56116.46

[B19] Collins JW Smith GS Baker SP Warner M Injuries related to forklifts and other powered industrial vehicles in automobile manufacturing American Journal Of Industrial Medicine 1999 36 5 513 21 10.1002/(SICI)1097-0274(199911)36:5<513::AID-AJIM3>3.0.CO;2-K 10506733

[B20] Lombardi DA Pannala R Sorock GS Wellman H Courtney TK Verma S Welding related occupational eye injuries: a narrative analysis Injury Prevention: Journal Of The International Society For Child And Adolescent Injury Prevention 2005 11 3 174 9 15933411 10.1136/ip.2004.007088 PMC1730216

[B21] Warner M Baker SP Li G Smith GS Acute traumatic injuries in automotive manufacturing American Journal Of Industrial Medicine 1998 34 4 351 8 10.1002/(SICI)1097-0274(199810)34:4<351::AID-AJIM8>3.0.CO;2-V 9750941

[B22] Bunn TL Slavova S Hall L Narrative text analysis of Kentucky tractor fatality reports Accident; Analysis And Prevention 2008 40 2 419 25 10.1016/j.aap.2007.07.010 18329390

[B23] Dement JM Lipscomb H Li L Epling C Desai T Nail gun injuries among construction workers Appl Occup Environ Hyg 2003 18 5 374 83 10.1080/10473220301365 12746081

[B24] Smith GS Timmons RA Lombardi DA Mamidi DK Matz S Courtney TK Work-related ladder fall fractures: identification and diagnosis validation using narrative text Accident; Analysis And Prevention 2006 38 5 973 80 10.1016/j.aap.2006.04.008 16750154

[B25] Williamson A Feyer AM Stout N Driscoll T Usher H Use of narrative analysis for comparisons of the causes of fatal accidents in three countries: New Zealand, Australia, and the United States Injury Prevention: Journal Of The International Society For Child And Adolescent Injury Prevention 2001 7 Suppl 1 i15 20 11565965 10.1136/ip.7.suppl_1.i15 PMC1765408

[B26] Jones SJ Lyons RA Routine narrative analysis as a screening tool to improve data quality Injury Prevention: Journal Of The International Society For Child And Adolescent Injury Prevention 2003 9 2 184 6 12810750 10.1136/ip.9.2.184 PMC1730971

[B27] Lincoln AE Sorock GS Courtney TK Wellman HM Smith GS Amoroso PJ Using narrative text and coded data to develop hazard scenarios for occupational injury interventions Injury Prevention: Journal Of The International Society For Child And Adolescent Injury Prevention 2004 10 4 249 54 15314055 10.1136/ip.2004.005181 PMC1730109

[B28] Bondy J Lipscomb H Guarini K Glazner JE Methods for using narrative text from injury reports to identify factors contributing to construction injury American Journal Of Industrial Medicine 2005 48 5 373 80 10.1002/ajim.20228 16254951

[B29] Glazner J Bondy J Lezotte DC Lipscomb H Guarini K Factors contributing to construction injury at Denver International Airport American Journal Of Industrial Medicine 2005 47 1 27 36 10.1002/ajim.20108 15597363

[B30] Lipscomb HJ Glazner J Bondy J Lezotte D Guarini K Analysis of text from injury reports improves understanding of construction falls Journal Of Occupational And Environmental Medicine/American College Of Occupational And Environmental Medicine 2004 46 11 1166 73 15534504 10.1097/01.jom.0000141769.48553.1b

[B31] Brooks B Shifting the focus of strategic occupational injury prevention: Mining free-text, workers compensation claims data Safety Science 2008 46 1 1 21 10.1016/j.ssci.2006.09.006

[B32] National Injury Surveillance Unit National Minimum Data Set for Injury Surveillance. Version 2.1 1998 Adelaide: National Injury Surveillance Unit

[B33] Leximancer White Paper 2009 https://www.leximancer.com/science/[updated 2009; cited 16 April 2009];

[B34] Smith A Humphries M Evaluation of unsupervised semantic mapping of natural language with Leximancer concept mapping Behavior Research Methods 2006 38 262 79 16956103 10.3758/bf03192778

